# Engineering the fatty acid synthesis pathway in *Synechococcus elongatus* PCC 7942 improves omega-3 fatty acid production

**DOI:** 10.1186/s13068-018-1243-4

**Published:** 2018-09-05

**Authors:** María Santos-Merino, M. Pilar Garcillán-Barcia, Fernando de la Cruz

**Affiliations:** Instituto de Biomedicina y Biotecnología de Cantabria (Universidad de Cantabria—Consejo Superior de Investigaciones Científicas), Santander, Cantabria Spain

**Keywords:** Cyanobacteria, Fatty acid synthesis, *fab* genes, Omega-3 fatty acids, *Synechococcus elongatus* PCC 7942

## Abstract

**Background:**

The microbial production of fatty acids has received great attention in the last few years as feedstock for the production of renewable energy. The main advantage of using cyanobacteria over other organisms is their ability to capture energy from sunlight and to transform CO_2_ into products of interest by photosynthesis, such as fatty acids. Fatty acid synthesis is a ubiquitous and well-characterized pathway in most bacteria. However, the activity of the enzymes involved in this pathway in cyanobacteria remains poorly explored.

**Results:**

To characterize the function of some enzymes involved in the saturated fatty acid synthesis in cyanobacteria, we genetically engineered *Synechococcus elongatus* PCC 7942 by overexpressing or deleting genes encoding enzymes of the fatty acid synthase system and tested the lipid profile of the mutants. These modifications were in turn used to improve alpha-linolenic acid production in this cyanobacterium. The mutant resulting from *fabF* overexpression and *fadD* deletion, combined with the overexpression of *desA* and *desB* desaturase genes from *Synechococcus* sp. PCC 7002, produced the highest levels of this omega-3 fatty acid.

**Conclusions:**

The fatty acid composition of *S. elongatus* PCC 7942 can be significantly modified by genetically engineering the expression of genes coding for the enzymes involved in the first reactions of fatty acid synthesis pathway. Variations in fatty acid composition of *S. elongatus* PCC 7942 mutants did not follow the pattern observed in *Escherichia coli* derivatives. Some of these modifications can be used to improve omega-3 fatty acid production. This work provides new insights into the saturated fatty acid synthesis pathway and new strategies that might be used to manipulate the fatty acid content of cyanobacteria.

**Electronic supplementary material:**

The online version of this article (10.1186/s13068-018-1243-4) contains supplementary material, which is available to authorized users.

## Background

There is an ample interest in turning cyanobacteria into biosynthetic platforms [1–3]. The main advantage of photosynthetic microorganisms is their capacity to economically transform sunlight and CO_2_ into value-added products without requiring organic compounds as raw materials. Therefore, the cyanobacterial production of fatty acids (FAs) has received great attention in the last few years as a sustainable feedstock for the production of high-energy–density biofuels and other FA-derived oleochemical compounds [4, 5].

The main FA synthesis pathway is highly conserved throughout bacterial species, and even in all kingdoms of life. Its importance comes from the fact that it is crucial for the cells, as it constitutes the first step in the formation of membrane lipids. The lipid composition of bacterial membranes is modified in response to environmental cues, allowing bacteria to survive under unfavorable conditions [6, 7]. In cyanobacteria, FAs are doubly important, since they are constituents of glycoglycerolipids, which form the thylakoid membranes, where photosynthesis takes place [8]. FA composition varies among cyanobacteria. This led Murata et al. [9] to classify them into four groups, a scheme recently updated to five [10]. Members of Group 1, such as *Synechococcus elongatus* PCC 7942 (Se7942), encode the Δ9 desaturase gene *desC* and contain saturated and mono-unsaturated FAs while lack polyunsaturated FAs. A richer FA composition can be found in other cyanobacteria, such as Group 3α *Synechococcus* sp. PCC 7002 (Ss7002), characterized by the production of trienoic alpha-linolenic acid (ALA, C18:3Δ^9,12,15^) by Δ9 (DesC), Δ12 (DesA) and Δ15 (DesB) desaturases.

**Fig. 1:**
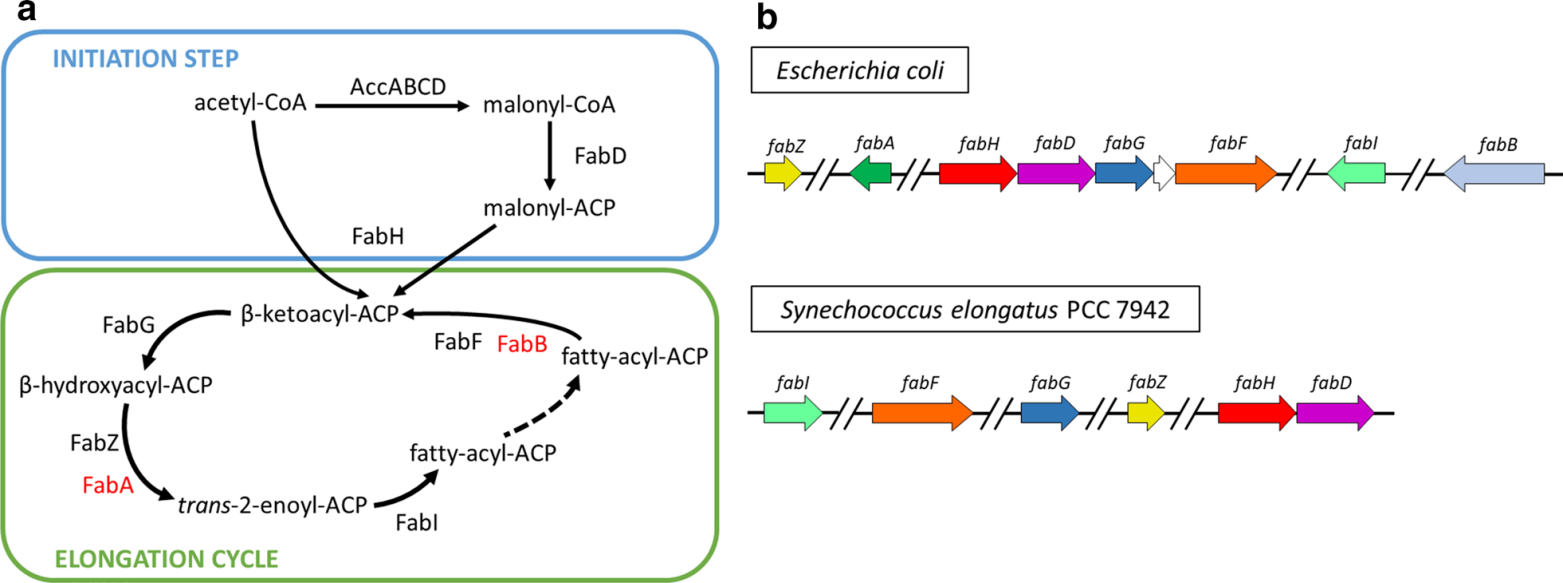
FA synthesis in *E. coli* vs Se7942 (**a**). Main FA synthesis pathway. In the initiation step, acetyl-CoA is converted into malonyl-CoA by the acetyl-CoA–carboxylase complex (AccABCD). Malonyl-CoA is transferred to the acyl-carrier protein (ACP) by FabD. Malonyl-CoA is condensed with acetyl-CoA by the β-ketoacyl-ACP synthase III (FabH). The resulting β-ketoacyl-ACP enters the elongation cycle, leading to long-chain acyl-ACP by serial steps of reduction (catalyzed by FabG), dehydration (FabZ and FabA), reduction (FabI), and elongation by condensing additional malonyl-ACP molecules (FabB and FabF). Enzymes present in *E. coli* and absent in Se7942 are colored in red. **b** Scheme of *fab* pathway gene organization in *E. coli* (upper panel) and Se7942 (lower panel). Homologous genes (or predicted ORFs) are depicted by arrows in the same color. The white arrow represents a gene not included in the *fab* cluster. Double slashes represent DNA regions that are not shown

FA synthesis has been widely studied in *Escherichia coli*, which serves as a general model for FA production in other bacterial species [11, 12]. FA synthesis requires two enzyme complexes, acetyl-CoA carboxylase (AccABCD), which catalyzes the committed step at the beginning of the FA pathway (conversion of acetyl-CoA into malonyl-CoA), and the FA synthase (FAS) system that catalyzes the remaining reactions [12]. Based on the organization of their catalytic units, there are two groups of FAS. Independently of the architecture of the FAS system, FA synthesis involves two steps, initiation and elongation [13] (Fig. 1a). Type I FAS carries out all steps of FA biosynthesis as a multimeric protein complex and is present in fungi and yeast genomes [14]. Type II FA synthases catalyze these reactions in most bacteria and consist of a series of enzymes, encoded by individual genes [15] (Fig. 1b). In many bacteria, such as *E. coli*, genes encoding core enzymes of the FAS system are organized in the *fab* (FA biosynthesis) cluster [16] (Fig. 1b, upper panel). On the contrary, in the case of Se7942, *fab* genes are non-clustered and rather scattered along the chromosome (Fig. 1b, lower panel). Moreover, the organization and distribution of the *fab* genes along the chromosome are different in both microorganisms. The composition of *E. coli* and Se7942 *fab* genes is similar except for the absence of the *fabA* and *fabB* in Se7942.

The enzymes involved in the FA synthesis pathway display high amino acid similarity between cyanobacterial and *E. coli* homologs [17, 18]. In fact, individual subunits of the Ss7002 and *E. coli* FAS complexes were qualitatively interchangeable in vitro, using purified protein components [17]. Nevertheless, the turnover rate of Ss7002 and *E. coli* FAS differed in the respective reconstituted systems. The metabolic flux of the Ss7002 FAS system was exclusively limited by FabH, a β-ketoacyl-ACP synthase that initiates FA synthesis by condensing malonyl-ACP with acetyl-CoA to form acetoacetyl-ACP (Fig. 1a) [17]. In contrast, the reconstituted *E. coli* system was limited by its dehydratase (FabZ) and enoyl reductase (FabI) [19]. Congruently, when the *fabZ*, *fabI* and *fabG* reductase genes were overexpressed in vivo, the resulting *E. coli* strain produced 50% more FAs [19]. Overproduction of FAs of different chain lengths from C6 to C16 was also obtained in *E. coli*, based on in silico predictive models of metabolic flux, when the *fabD* strain was engineered to overproduce the *fabZ* and acyl-ACP thioesterase genes [20]. Finally, the abundance of FA synthesis proteins was compared between a FA high-yield *E. coli* strain (*fadE* deficient) and the wild-type (wt) strain by mass spectrometry [21]. Higher levels of acetyl-CoA carboxylase, thioesterase and FabZ were detected in the FA high-yield strain, implying that they are key enzymes for improving FA production in *E. coli*.

In this work, we genetically modified the expression of several enzymes involved in the FA synthesis pathway in the model cyanobacterium Se7942 and evaluated their impact on the lipid profile. Our results showed remarkable differences in the functionality of some of these enzymes between Se7942 and *E. coli*. This analysis allowed the selection of genetic modifications that, combined with the introduction of Ss7002 genes coding for DesA and DesB desaturases, rendered an engineered Se7942 strain able to produce ALA up to 22.6% of its total FA profile. These modifications might be implemented in other cyanobacteria, especially in omega-3 natural producers, to increase their yield.

## Results

### Overproduction of enzymes involved in the initiation step of fatty acid synthesis pathway changes the lipid profile of Se7942

**Fig. 2:**
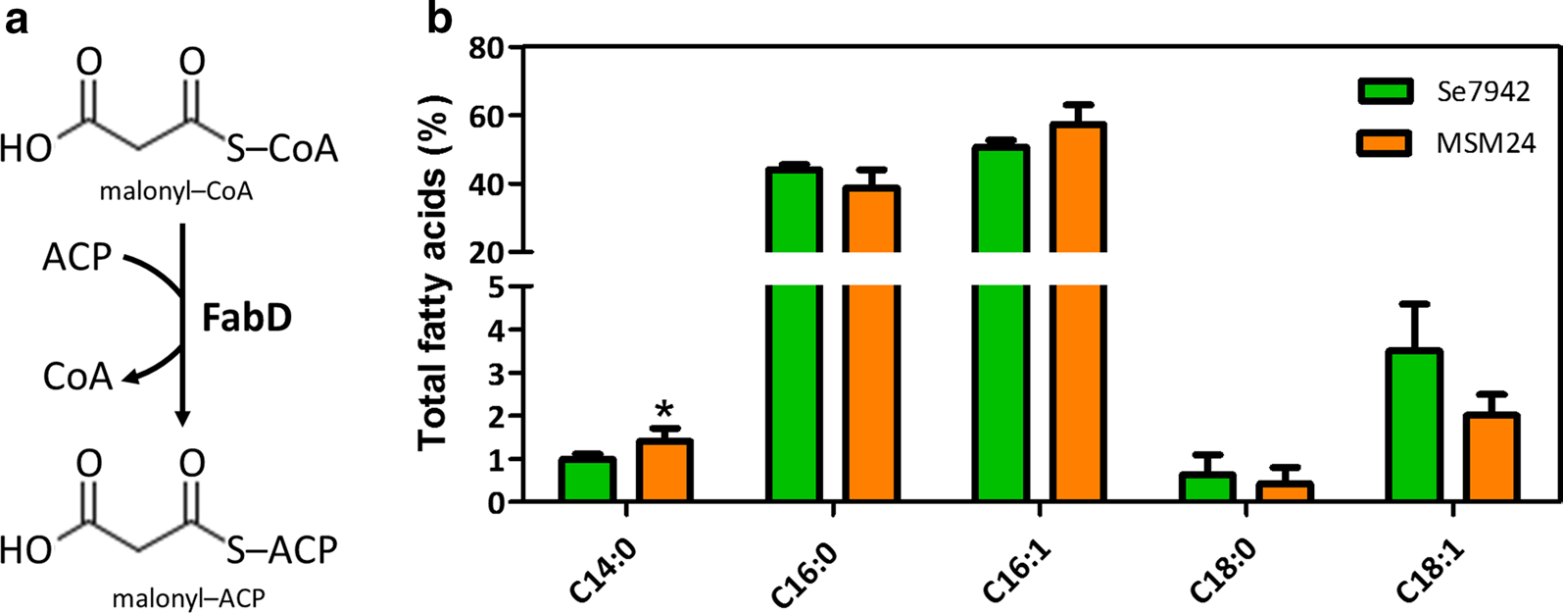
Effect of *fabD* overexpression in FA composition. **a** Enzymatic reaction catalyzed by FabD. **b** FA content of wt Se7942 and the *fabD* mutant derivative, MSM24. The different FA species are shown along the *x*-axis. The y-axis shows the percentage of each FA with respect to the total amount of FAs analyzed. Data are the average of at least three independent biological replicates and are represented as the mean + SD. *p < 0.05, by unpaired Student’s *t* test

FabD, the first enzyme of the *fab* cluster involved in saturated FA synthesis, is a malonyl-CoA ACP transacylase (MCAT). It catalyzes the malonyl-CoA transfer to ACP, generating malonyl-ACP and free CoA [22] (Figs. 1a, 2a). *fabD* overexpression in *E. coli* produced an altered FA composition, increasing the proportion of *cis*-vaccenic acid (C18:1n7) while decreasing palmitoleic acid (C16:1) [23]. To test if FabD overproduction in Se7942 had the same effect, strain MSM24, containing a P*trc*::*fabD* cassette in neutral site 1 (NS1), was constructed. As shown in Fig. 2b, the FA profile exhibited small changes in C14:0 and C18:1, but only in the case of C14:0 this change was statistically significant. In *E. coli*, changes in C14:0 were not observed, while an increase in C18:1 was detected as a consequence of a concomitant decrease in C16:1 [23]. These results demonstrated that *fabD* overexpression did not produce the same effect in both bacteria.

**Fig. 3:**
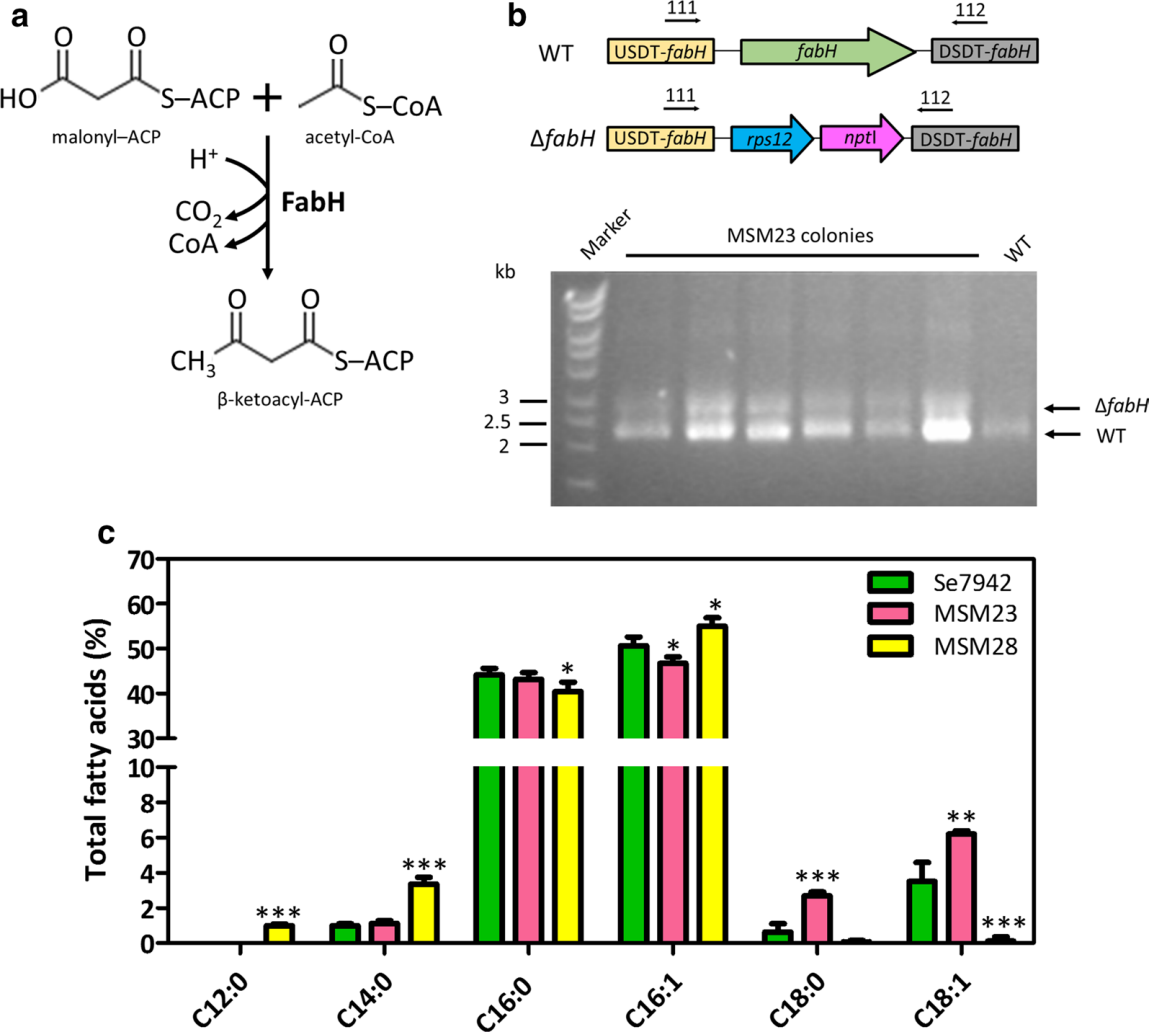
Effects of FabH levels on Se7942 FA content. **a** Enzymatic reaction catalyzed by FabH. **b** Attempt to construct the Se7942 *fabH*-deficient mutant. Upper panel: scheme of the *fabH* vicinity in the wt and *fabH* mutant strains. Relevant genes are indicated by colored arrows, while upstream and downstream sequences flanking the deletion target (USDT and DSDT) by boxes. Primers used for PCR analysis are indicated by black arrows. Lower panel: PCR analysis of the *fabH* region of mutant strain MSM23. Lane 1, Hyperladder I (BioLine); lanes 2–7, MSM23 colonies; lane 8, wt Se7942 strain (WT). **c** FA content of wt Se7942 and the *fabH* merodiploid mutant (MSM23) and *fabH*-overexpressing derivative (MSM28). Data show the mean and + SD of at least three independent biological replicates. *p < 0.05, **p < 0.01, ***p < 0.001, by unpaired Student’s *t* test

The next target in the FA synthesis pathway to be modified in this study was the β-ketoacyl-ACP synthase III (KAS III), which catalyzes the formation of 3-ketoacyl-ACP by condensation of acetyl-CoA with malonyl-ACP (Fig. 3a). This enzyme is encoded by the *fabH* gene and produces the precursor needed for the elongation cycle [24] (Fig. 1a). Subsequent elongation steps are performed by FabB and FabF, described in more detail below.

In *E. coli*, *fabH* deletion improved the production of C18 species, while reducing C16 [25]. Overexpression of *fabH* had an opposite effect: C14 and C16 increased at the expense of C18:1 [26]. In Ss7002, Kuo and Khosla determined that FabH was the rate-limiting enzyme of FAS using in vitro reconstituted systems and suggested that, either overexpressing the endogenous *fabH* gene or by replacement with its *E. coli* ortholog, the FA flux should be increased [17]. Attempts to delete *fabH* in Ss7002 proved impossible, rendering always non-pure deficient mutants and thus suggesting that *fabH* is essential in this cyanobacterium [27].

Based on this information, two different modifications were carried out in Se7942: a mutant that overexpressed *fabH* under the P*trc* promoter (MSM28), and a merodiploid mutant in which some chromosomal copies of the *fabH* gene were deleted (MSM23). To generate the latter, we used the gene replacement system designed by Matsuoka et al. [28]. A kanamycin resistance cassette and a wt copy of the *rps12* gene of *Synechocystis* sp. PCC 6803 (Ss6803) were inserted into the Se7942 *fabH* open reading frame (Fig. 3b, upper panel). After purification of the transformant colonies by successive streakings, the *fabH* mutant was checked by PCR using primers 111 and 112 (Additional file 3: Table S3). Two amplicon bands were observed in the electrophoresis gel. The largest band (~ 2.6 kb) belonged to the chromosomes with the *fabH* deletion and the smallest one (~ 2.1 kb) to the wt chromosome (Fig. 3b, lower panel). Using a transposon mutagenesis approach, *fabH* was described as an essential gene in Se7942 [29], a probable reason for obtaining here only merodiploid cells that contained both inactivated and *fabH* wt copies. On the other hand, we were able to delete this gene in Se7942 only when an extra copy of the *fabH* gene was previously inserted under the control of a strong constitutive promoter, P*trc* (strain MSM34, Additional file 1: Figures S1 and S2B). Lower expression of the extra copy of *fabH*, driven by a weaker promoter P*nrsB* with a theophylline-dependent riboswitch [30] in the absence of inducers, did not allow complete deletion of the wt gene (strain MSM35, Additional file 1: Figure S1 and S2C).

The *fabH* mutants were used to compare their FA composition with the wt strain (Fig. 3c). Lauric acid (C12:0) was detected only in MSM28, the strain that overexpresses *fabH*. FabH overproduction resulted also in an increase in medium-chain FAs (MCFAs) (C12:0 and C14:0) and a decrease in long-chain FAs (LCFAs) (C18:0 and C18:1), as happened in *E. coli* [26]. In contrast, the merodiploid mutant, MSM23, showed higher production of LCFAs (C18 and C18:1) and lower yield of C16:1, also similar to the FA profile described for the *E. coli fabH* derivative [25].

### FabF overproduction increases transformation of C14 and C16 fatty acids into C18

**Fig. 4:**
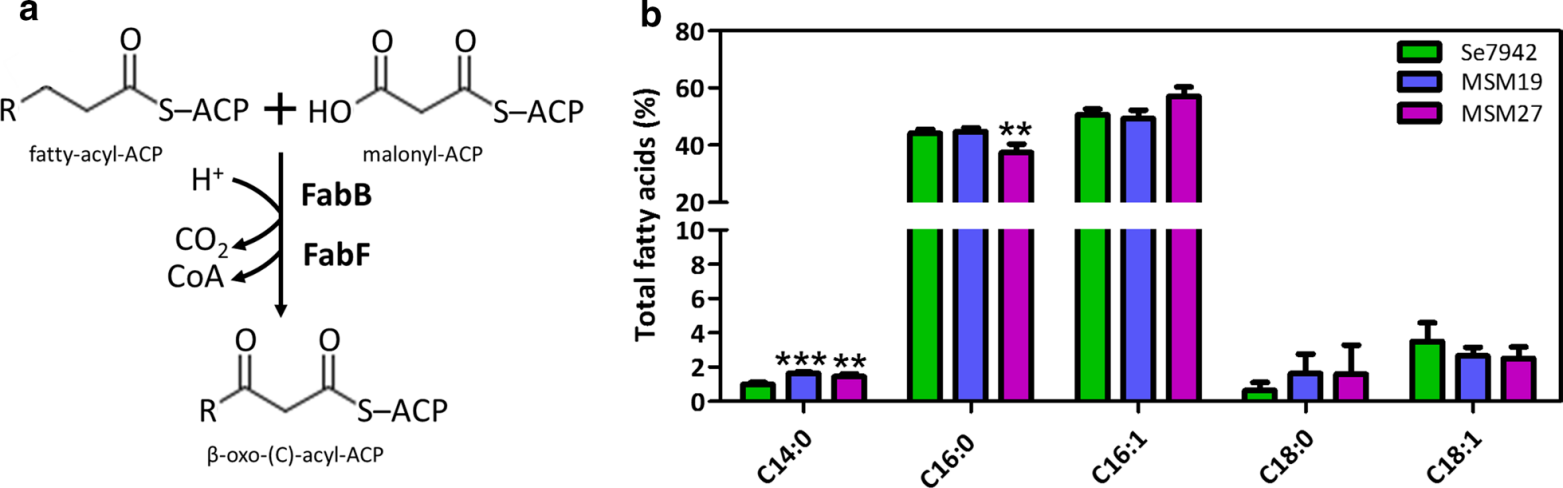
Effect of the *fabB* overexpression on FA composition. **a** Enzymatic reaction catalyzed by FabB and FabF. The KASs type I and II, FabB and FabF, catalyze the Claisen condensation of FA-thioesters and malonyl-ACP to form a β-ketoacyl-ACP intermediate elongated by two carbon atoms (β-oxo-(C)-acyl-ACP). “R” represents the hydrocarbon chain and “(*C*)” the number of carbon present in the FA. **b** FA content of wt Se7942 (green bars) and the *fabB* overexpressing mutants MSM19 (blue) and MSM27 (purple). Data are the average of at least three independent biological replicates and are represented as the mean + SD. **p < 0.01, ***p < 0.001, by unpaired Student’s *t* test

FabB and FabF, both independently catalyze the first reaction in the second elongation cycle (Fig. 4a). FabB is a KAS I, which catalyzes the condensation of cis-3-decenoyl-ACP, cis-5-dodecenoyl-ACP, and cis-7-tetradodecenoyl-ACP with malonyl-ACP [31]. In *E. coli*, FabB showed activity with saturated C4–C14 FAs as substrates and was involved in the control of unsaturation [32]. Furthermore, *fabB* overexpression increased the amount of C18:1 [33]. The *fabB* gene is present in Ss7002 but no homolog is found in Se7942. Kuo and Khosla determined that *fabB* was not essential in Ss7002 and speculated that it could play an additional role in fast-growing Ss7002 [17]. Based on these results, we tried to identify the function of this gene by analyzing the effects of its overexpression in Se7942.

To overproduce FabB in Se7942, the Ss7002 *fabB* gene was cloned under P*nrsB* and P*trc* promoters, generating strains MSM19 and MSM27, respectively (see Additional file 2 for details). As shown in Fig. 4b, FabB overproduction did not lead to major changes in the FA profile. The most significant variation was the increase in C14:0. This fact suggests that FabB has more affinity for MCFAs, as happens in *E. coli* [32]. On the other hand, in MSM27 the amount of C16:0 slightly diminished (Fig. 4b, purple bar). This effect was not observed in MSM19, which carries a weaker promoter to produce FabB than MSMS27, and consequently, it could be related with the amount of FabB protein obtained during overexpression (Additional file 1: Figures S2A and S2B, respectively).

**Fig. 5:**
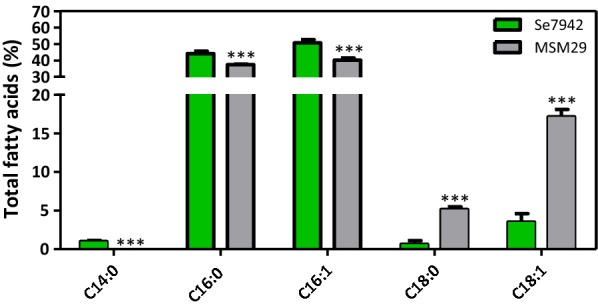
Effect of the *fabF* overexpression on FA composition. FA content of wt Se7942 (green bars) and the *fabF* mutant derivative, MSM29 (gray). Data are the average of at least three independent biological replicates and are represented as the mean + SD. ***p < 0.001, by unpaired Student’s *t* test

FabF also catalyzes the reaction shown in Fig. 4a. FabF is a KAS II and catalyzes the same condensation reaction as KAS I enzymes. In *E. coli*, KAS II is predominantly responsible for the elongation of palmitoleic acid (C16:1) required for the *cis*-vaccenic acid (C18:1n-7) synthesis [32]. In *E. coli*, *fabF* overexpression is lethal, as high levels of FabF block the access of FabB to the product generated by FabD [34]. Due to the lack of *fabB* in Se7942, this reaction is only catalyzed by FabF, and thus the blocking effect observed in *E. coli* should not take place. To verify this end, we constructed Se7942 mutant MSM29, which overexpresses *fabF*. This mutant showed a significant decrease in MCFAs (C14:0, C16:0 and C16:1) linked to a significant increase in LCFAs (C18:0 and C18:1) (Fig. 5). These results indicate that high FabF levels improved the transformation of C14 and C16 into C18.

### FadD deletion increases transformation of C16:1 into C18

**Fig. 6:**
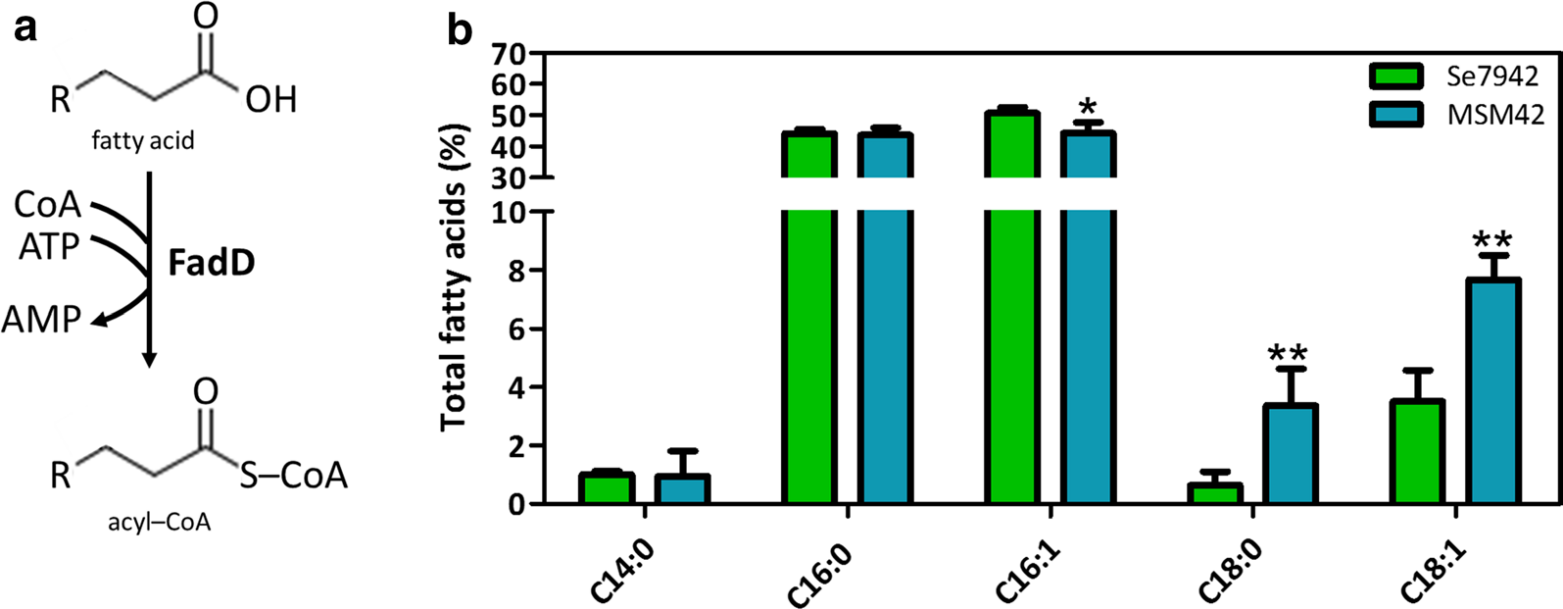
Effect of the *fadD* deletion on FA composition. **a** Enzymatic reaction catalyzed by FadD. FadD is an acyl-CoA synthetase that loads free FAs on CoA. “R” represents the hydrocarbon chain. **b** FA content of wt Se7942 (green bars) and the *fadD* mutant derivative, MSM42 (cyan bars). Data are the average of at least three independent biological replicates and are represented as the mean + SD. *p < 0.05, **p < 0.01, by unpaired Student’s *t* test

The metabolic pathway to degrade MCFAs and LCFAs is called β-oxidation. Despite being highly conserved, it is not present in cyanobacteria, according to the literature and genome annotation. The first step in this pathway is catalyzed by FadD, a long-chain fatty acyl-CoA ligase (acyl-CoA synthetase) responsible for the activation of exogenous LCFAs into acyl-CoAs [35] (Fig. 6a). FadD is the single gene of this pathway identified in cyanobacteria [36]. In *E. coli*, *fadD* disruption caused the accumulation of free FAs in the cytosol [35, 37]. In Se7942, *fadD* deletion produced a change in the FA profile of membrane lipids, particularly an increase in the degree of saturation [38].

Based on this information, we disrupted the *fadD* gene in Se7942, obtaining strain MSM42 (Additional file 1: Figure S3A), which is a pure *fadD* mutant (Additional file 1: Figure S3B). The lipid profiles of the engineered and wt strains were analyzed and compared. As shown in Fig. 6b, MSM42 exhibited a significant increase in C18 FAs (C18:0 and C18:1) at the expense of C16:1 content decrease.

### Overexpression of *desA* and *desB* desaturase genes leads to increase alpha-linolenic acid production at low temperature

**Fig. 7:**
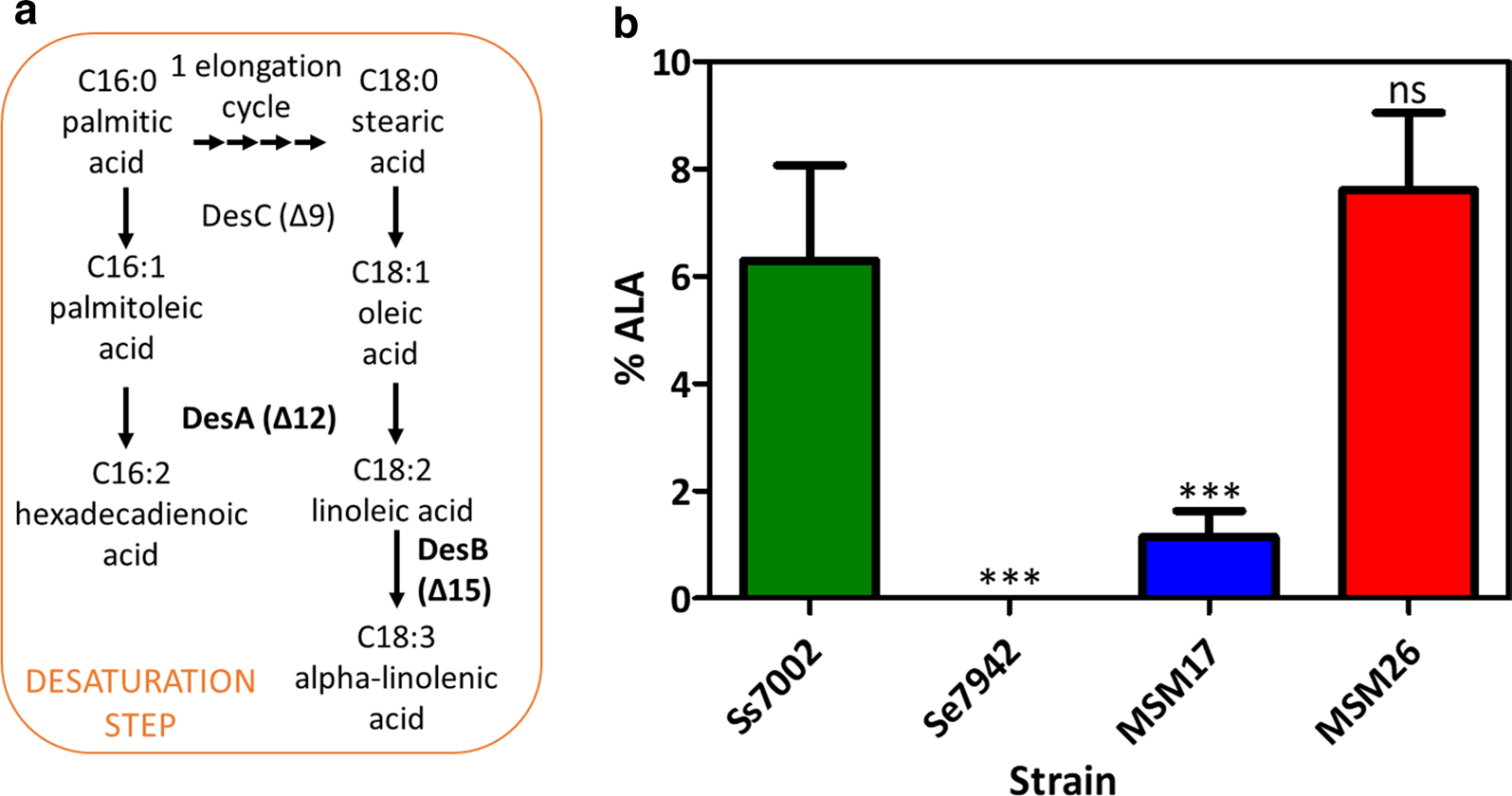
ALA production in Se7942. **a** Desaturation reactions needed to produce ALA in cyanobacteria. In bold, desaturase genes introduced in Se7942. **b** ALA content of wt Ss7002 (green bar), wt Se7942, *desA* and *desB* mutant derivatives, MSM17 (blue) and MSM26 (red), respectively. Data are the average of at least three independent biological replicates and are represented as the mean + SD. ***p < 0.001, by one-way analysis of variance (ANOVA) followed by Dunnett’s multiple comparison test with a single control, Ss7002

Three desaturases are essential to produce ALA in cyanobacteria, DesC, DesA and DesB (Fig. 7a). DesC introduces the first *cis* double bond in the Δ9 position of stearic acid (C18:0), producing oleic acid (C18:1). The next double bond is introduced at the Δ12 position of C18:1 by DesA, producing linoleic acid (C18:2). Finally, DesB introduces a third double bond at the Δ15 (ω3) position of C18:2, rendering ALA. DesC and DesB may also use C16 FAs as substrates [39, 40]. Se7942 encodes *desC* but lacks both *desA* and *desB* desaturase genes [41], whose role in ALA production is well documented in Ss7002 [42]. The *desA* mRNA accumulates faster in Ss7002 [43] than in other cyanobacteria, such as Ss6803 [10]. Thus, *desA* and *desB* from Ss7002 were chosen as candidate genes to be introduced in Se7942 for producing ALA in this organism.

Two Se7942 derivative strains were constructed by integrating Ss7002 *desA* and *desB* genes in the NS1 site: MSM17 (P*nrsB*::*desA*-*desB*) and MSM26 (P*trc*::*desA*-*desB*). Optimal conditions to express these genes were previously defined in Ss7002. Both *desA* and *desB* transcripts were more abundant at low temperature, specifically at 22 °C [44, 45]. Therefore, ALA production in Se7942 was tested at this temperature. Both mutant strains produced ALA (Fig. 7b). In the case of MSM17, which expresses *desAB* genes from the P*nrsB* promoter, ALA production (1.14% of total lipids) was significantly lower than that of Ss7002. Replacement of P*nrsB* by a stronger promoter, P*trc* (strain MSM26), led to an increase of approximately 6.6 times in ALA production yield, reaching levels as high as those obtained from Ss7002.

Based on data recently published by Yang et al. [46] in the Cyanomics database, a condition that might improve the expression of both desaturases, i.e., high light intensity, was identified. This database comprises genomic, transcriptomic and proteomic datasets of Ss7002. This condition had been already tested by Sakamoto et al., who found an increase of 3.8 times in ALA production by Ss7002 under high light intensity (250 µmol photons m^−2^ s^−1^) [43]. The influence of light intensity on ALA production was thus investigated in the engineered Se7942 strain MSM26. No statistically significant difference was observed under low light intensity (60 µmol photons m^−2^ s^−1^), 7.61 ± 1.45%, versus 8.78 ± 1.79% under high light intensity (250 µmol photons m^−2^ s^−1^).

### Overproduction of FabF and DesAB in a Se7942 *fadD* deletion mutant improves alpha-linolenic acid yields

**Fig. 8:**
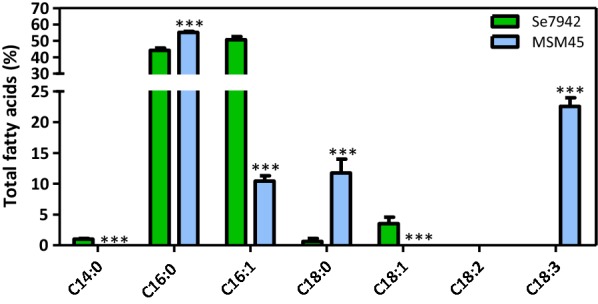
Effect of ALA production in the FA Profile. wt Se7942 and the ALA-producing mutant, MSM45, are represented by green and blue bars, respectively. Data are the average of at least three independent biological replicates and are represented as the mean + SD. ***p < 0.001, by unpaired Student’s *t* test

By construction of a series of Se7942 mutants, we observed that both *fabF* overexpression and *fadD* deletion improved the ALA production, whereas *fabH* partial deletion did not (data not shown). The combined effect of both modifications was checked in the mutant strain MSM45, which lacked *fadD* and overexpressed *fabF* and both desaturase genes (*desA* and *desB*). We compared the FA composition of MSM45 versus wt Se7942 and several differences were observed (Fig. 8). First, a significant reduction in C14:0, C16:1 and C18:1 was produced in MSM45, as well as a significant increase in C16:0 and C18:0. Strain MSM45 produced ALA to as high levels as 22.6%. This amount was higher than the ALA production by Ss7002 under high light condition (19%) [43].

## Discussion

Se7942 is a model cyanobacterium because of its small genome size and simple metabolism, making it ideal for the study of lipid biosynthesis in photoautotrophic bacteria. To gain a detailed understanding of the FA synthesis pathway in Se7942, a deeper knowledge on the activity of the enzymes involved was needed. To fulfill this aim, we engineered several modifications in the production of these enzymes in Se7942. Our results showed important differences in the activity of some enzymes involved in FA synthesis in Se7942 regarding those in *E. coli*, despite their amino acid sequence homology (ranging from 42 to 52% identity). Whereas *fabD* overexpression altered the FA composition of *E. coli* [23], this modification did not have a remarkable effect in the Se7942 FA profile. On the other hand, the *E. coli fabH* mutant is viable and exhibits a small-colony phenotype [25], suggesting the implication of another condensing enzyme performing less efficiently this reaction in the initiation cycle of the FA biosynthesis. In Se7942, the *fabH* deletion was lethal, when assayed by random barcode transposon site sequencing (RB-TnSeq) [29]. Neither was this gene removed from all chromosomal copies of Ss7002 [27]. Our attempts to delete *fabH* in Se7942 also resulted in merodiploid mutants, an indication of the indispensability of this gene, and that no other Se7942 KAS enzyme (e.g., FabF) can act as a functional replacement for FabH. Overexpression of *fabH* led to increase in MCFA production, specifically lauric (C12:0) and myristic (C14:0) acids. Notably, C12:0 is not naturally present in Se7942. Increased levels of MCFAs were reported in an engineered Ss7002 strain in which *fabH* was replaced by *Chaetoceros* GSL56 *KASIII* gene [27]. MCFAs are appreciated in the fuel industry, which makes *fabH* overexpression in cyanobacteria potentially useful. These results suggest that in cyanobacteria FabH may have more affinity toward short-chain FAs, a feature described previously in other bacteria [47].

Regarding the two other KAS, FabB and FabF, relevant differences to *E. coli* were found. In *E. coli*, FabB plays an important role in controlling the unsaturation degree of FAs [33]. It is noteworthy that FabB is absent in Se7942 and most cyanobacteria, but present in Ss7002, where it is dispensable in unsaturated FA synthesis [17]. In cyanobacteria, unsaturated FA synthesis is controlled downstream of the FA synthases, because their synthesis is performed by desaturases, which act on FAS products [40]. The growth temperature of cyanobacteria is responsible for the up- or downregulation of the desaturase gene expression [48]. Overproduction of FabB from Ss7002 in Se7942 did not increase C18:1 yield, contrary to the *E. coli* case [33], suggesting that FabB did not play a role in the cyanobacterial unsaturated FA synthesis. Kuo and Khosla speculated that FabB could have an additional role, still unknown [17]. Overexpression of *fabF* was lethal in *E. coli*, likely due to the fact that high levels of FabF block the access of FabB to the product generated by FabD [34]. This lethality was not observed when the endogenous *fabF* gene was overexpressed in Se7942, ruled out any FabB blockage by FabF since FabB is absent in Se7942.

Taking all these results together, we suggest that the enzymes involved in FA synthesis in Se7942 play a different role from that observed in *E. coli*. Moreover, it also suggests that these enzymes could be regulated in a different way in both bacteria. Even though the biochemistry of FA synthesis in *E. coli* is well known and documented, its genetic regulation still remains unclear. In *E. coli*, the key regulators are the long-chain acyl-ACP end products, which exert a negative regulatory feedback on key enzymes of the synthesis pathway [49]. Moreover, this pathway is also controlled by transcription factors, mainly FabR (TetR family) and FadR (GntR family), among others [49]. FabR and FadR also control the expression of FA degradation genes involved in the β-oxidation cycle [50]. As mentioned above, it appears that cyanobacteria lack the major FA degradative metabolic pathway β-oxidation, generally thought to be universal. In several Gram-positive bacteria, there are other families of regulators that control the expression of *fab* genes. In *Bacillus subtilis*, FapR is a global negative regulator of genes involved in FA and phospholipid biosynthesis [51]. This regulator, a member of the DeoR family, is common to other species of the *Bacillus* genus, as well as *Listeria*, *Clostridium* and *Staphylococcus* [51] and senses malonyl-CoA, which releases its transcriptional repression [52, 53]. In *Streptococcus pneumoniae*, a different FA synthesis regulator was identified, FabT, which belongs to the MarR family [54]. FabT homologs were also located in other groups of Gram‐positive bacteria, such as *Enterococcus*, *Clostridium* and *Lactococcus* [54].

Little is known about the regulation of the gene expression of FA synthesis in cyanobacteria. In most bacteria, *fab* genes are organized into operons, and this organization enables the identification of cognate transcription factors based on shared synteny [55]. In cyanobacteria [56], *fab* genes are scattered throughout the genome. Moreover, due to the fact that cyanobacteria do not code for a FA degradation pathway, some authors suggested that the regulatory of mechanisms for FA metabolism may be largely different from those of heterotrophic bacteria [56, 57]. There is scarce information on the transcriptional regulation of FA biosynthesis in cyanobacteria. Deletion of a cyanobacteria-specific transcriptional regulator gene, *cyAbrB2*, was found to enhance the production of free FAs in engineered strains of Ss6803 [58]. Besides, the LexA transcription factor was described as a regulator of FA synthesis also in Ss6803, and more specifically as a repressor of genes involved in the initiation step of FA synthesis (*fabD*, *fabH* and *fabF*) and the first reductive step in the elongation cycle (*fabG*) [56]. Gene *lexA* deletion largely increased the production of FAs in an engineered Ss6803 strain [56]. No LexA homolog was found in Se7942 [59]. The information related to FA synthesis regulation in Se7942 focuses on the signal transduction protein P_II_. The P_II_ mutant strain of Se7942 showed a decreased intracellular acetyl-CoA content and enhanced activity of the acetyl-CoA–carboxylase complex through transcriptional activation of the *accABCD* genes [60]. However, P_II_ does not regulate the enzymes involved in the FAS system. These and our results may suggest that the regulation of FA synthesis enzymes in cyanobacteria is independent of FA degradation by the β-oxidation cycle and could involve a global regulator, which has yet to be found.

Omega-3 FAs are naturally produced in some cyanobacteria, members of the groups 3α and 4 [10], which encode Δ12 and Δ15 desaturase genes. Such is the case of Ss6803 [61], *Synechococcus* sp. NKBG 15041c [62] and Ss7002 [43]. The first two produced high levels of ALA (23 and 50% of total FA, respectively) when engineered to overexpress, respectively, Δ6 + Δ15 and Δ6 FA desaturase genes [63, 64]. On the other hand, Ss7002 produced the highest ALA levels (19% of total FA) in a cyanobacterial wt strain when grown at high light intensity either at 22 °C or at 38 °C and shifted to 22 °C for 12 h [43]. The introduction of the Δ12 desaturase gene *desA* from Ss6803 into Se7942 caused a modification of its FA composition: dienoic FA C16:2Δ^9,12^ and C18:2Δ^9,12^ emerged at significant levels at the expense of C16:1Δ^9^ and C18:1Δ^9^ [65]. This modification led to an enhancing in chilling tolerance [65] and protection against photoinhibition [66]. When transformed with Ss6803 Δ12 and Δ15 desaturase genes, *desA* and *desB*, Se7942 was able to desaturase FA at Δ9, Δ12 and Δ15 positions [67]. ALA production by the transformant strain at 34 °C was low (1% of total lipids) but significantly increased (up to 5%) when incubated at 22 °C. Taking advantage of the high similarity in codon usage between Se7942 and Ss7002, the Δ12 and Δ15 desaturase genes of the latter, *desA* and *desB*, were chosen to produce transgenically ALA in Se7942, which lacks these two essential enzymes of the desaturation pathway. The *desB* gene of Ss7002 is transcribed only at low temperatures (22 °C) [44, 45]. Thus, an inducible promoter was chosen to drive the *desAB* expression at 22 °C once enough biomass was achieved at 30 °C, because Se7942 growth is impaired at low temperature [39]. The resulting strain, MSM26, produced ALA at the same level of Ss7002 (Fig. 7b), at the expense of C18 and C16:1 FAs. Se7942 mutants either overexpressing *fabF* or lacking *fadD* exhibited a FA profile enriched in C18 and thus these mutations were evaluated in combination with *desAB* expression. This strategy resulted in a significant improvement in the yield of this omega-3 FA in relation to the available data for Ss7002 [43, 45, 68, 69], and comparable to the engineered Se6803 [63].

## Conclusion

In this study, the activity of the enzymes involved in the first reactions of FA synthesis pathway in Se7942 was examined by deleting or overexpressing their respective coding genes and analyzing the resulting FA profiles. In general, the mutant strains showed changes in their FA composition, regarding both the wt Se7942 strain and *E. coli* mutants. In addition, the modifications that improved the synthesis of C16:0 and C18:0 (overexpression of *fabF* and deletion of *fadD*) used in combination with the expression of Ss7002 *desAB* desaturase genes was revealed as a feasible strategy to engineer Se7942 for ALA production. To the best of our knowledge, this is the first report on increased ALA production in cyanobacteria using modifications in the expression of the genes belonging to the FAS system. The combination of *fadD* deletion and *fabF* overexpression is a strategy that may be evaluated in omega-3 natural producers to raise ALA yield.

## Methods

### Strains and culture conditions

All strains used in this study are listed in Table 1. Ss7002 was cultured in A + medium [70], while Se7942 and GRPS1 were cultured in BG11 medium [71]. All cyanobacterial strains were grown at 30 °C by bubbling 1% CO_2_ with continuous light at 60 µmol photons m^−2^ s^−1^. Antibiotics used for selecting Se7942 were neomycin at 5 or 25 µg/ml (Neo5 or Neo25), spectinomycin at 10 or 20 µg/ml (Sp10 or Sp20) and chloramphenicol at 5 or 10 µg/ml (Cm5 or Cm10).

**Table 1:** Cyanobacterial strains used in this study

Strain	Relevant genotype and phenotype^a^	Plasmids used to generate this strain^b^	Source or references
*Synechococcus* sp. PCC 7002	Wild-type strain		PCC
*Synechococcus elongatus* PCC 7942	Wild-type strain		PCC
GRPS1	*S. elongatus* PCC 7942 (*rps12*–*R43*); Sm^r^		[28]
MSM17	*S. elongatus* PCC 7942 with P*nrsB:*:*desA*–*desB* integrated at NS1; Sp^r^	pMSM16	This work
MSM18	*S. elongatus* PCC 7942 with P*nrsB*::*luxAB* integrated at NS1; Sp^r^	pMSM51	This work
MSM19	*S. elongatus* PCC 7942 with P*nrsB*::*fabB* integrated at NS1; Sp^r^	pMSM90	This work
MSM22	*S. elongatus* PCC 7942 with P*nrsB*::*riboswitch*–*luxAB* integrated at NS1; Sp^r^	pMSM142	This work
MSM23	GRPS1 with Δ*fabH*::*rps12*–*npt*I (partial deletion); Neo^r^	pMSM182	This work
MSM24	*S. elongatus* PCC 7942 with P*trc*::*fabD* integrated at NS1; Sp^r^	pMSM196	This work
MSM25	*S. elongatus* PCC 7942 with P*trc*::*luxAB* integrated at NS1; Sp^r^	pMSM197	This work
MSM26	*S. elongatus* PCC 7942 with P*trc*::*desA*–*desB* integrated at NS1; Sp^r^	pMSM201	This work
MSM27	*S. elongatus* PCC 7942 with P*trc*::*fabB* integrated at NS1; Sp^r^	pMSM202	This work
MSM28	*S. elongatus* PCC 7942 with P*trc*::*fabH* integrated at NS1; Sp^r^	pMSM228	This work
MSM29	*S. elongatus* PCC 7942 with P*trc*::*fabF* integrated at NS1; Sp^r^	pMSM234	This work
MSM34	GRPS1 with Δ*fabH*::*rps12*–*npt*I; Neo^r^ P*trc*::*fabH* integrated at NS1; Sp^r^	pMSM182 pMSM228	This work
MSM35	GRPS1 Δ*fabH*::*rps12*–*npt*I (partial deletion); Neo^r^ P*nrsB*::*riboswitch*–*fabH* integrated at NS1; Sp^r^	pMSM182 pMSM236	This work
MSM42	GRPS1 Δ*fadD*::*rps12*- *npt*I; Neo^r^	pMSM266	This work
MSM45	GRPS1 P*trc*::*desA*–*desB* integrated at NS1; Sp^r^ P*trc*::*fabF* integrated at NS2; Cm^r^ Δ*fadD*::*rps12*–*npt*I; Neo^r^	pMSM201 pMSM253 pMSM266	This work

*PCC* Pasteur Culture Collection

^a^Sm^r^, streptomycin resistance; Neo^r^, neomycin resistance; Sp^r^, spectinomycin resistance; Cm^r^, chloramphenicol resistance; NS1, neutral site 1; NS2, neutral site 2. Integration of vectors into NS1 and NS2 was verified by PCR using primer pairs 102–103 and 106–108, respectively

^b^Plasmids are listed in Additional file 3: Table S1 and, primers and details on their construction are described in Additional file 3: Table S2 and Additional file 2, respectively

*Escherichia coli* strain DH5α was used for transformation with recombinant plasmids. It was grown in Luria–Bertani (LB) medium at 37 °C under shaking (150 rpm) and, when required, was supplemented with antibiotics: kanamycin, 50 µg/ml (Km50), chloramphenicol, 25 µg/ml (Cm25), spectinomycin, 100 µg/ml (Sp100) and ampicillin, 100 µg/ml (Ap100).

### Natural transformation of Se7942

Transformation of Se7942 was performed as previously described [72]. Briefly, a Se7942 culture sample equivalent to 10 μg of chlorophyll (around 4 × 10^9^ cyanobacterial cells) was mixed with 500 ng plasmid DNA for 24 h in the dark at 30 °C. Transformation mixtures were deposited onto nitrocellulose filters (Millipore) and incubated for 24 h on BG11 plates at 30 °C with continuous light. Transformant colonies were selected in BG11 supplemented with the corresponding antibiotic. Mutant segregation was achieved by repeatedly transferring individual transformant colonies to fresh selective plates. Mutant genotypes were confirmed by PCR followed by DNA sequencing using specific primers (see Additional file 3: Tables S1 and S3).

### Cultivation of cyanobacteria and engineered strains for fatty acid production

Se7942 was cultured in BG11 medium in a Multi-cultivator MC 1000-OD (Photon Systems Instruments, Drasov, Czech Republic) with cool white light (60 µmol photons m^−2^ s^−1^) at 30 °C and 3% CO_2_. From solid BG11 agar medium, individual colonies were inoculated into 60 ml of BG11 and grown for 5–7 days to be used as inocula. Cultures were inoculated at initial OD_720_ = 0.05 in 60 ml of fresh BG11 medium (without antibiotics). At OD_720_ 0.5–0.6, the temperature was decreased from 30 °C to 22 °C, IPTG (P*trc* promoter) or NiSO_4_ (P*nrsB* promoter) was added to 1 mM or 5 µM final concentrations, respectively. Samples were taken 24 h post-induction.

### Chromosomal gene deletion system based on a dominant streptomycin-sensitive *rps12* gene

Gene deletion was carried out in the Se7942 derivative strain GRPS1 [28], using the *rps12*-mediated gene replacement method [73]. In this approach, a transformation is performed using a plasmid that contains *rps12* and *npt*I genes flanked by USDT (upstream sequences of the deletion target) and DSDT (downstream sequences of the deletion target). Transformant colonies were selected using Neo25. Individual colonies were checked for the Neo^r^/Sm^s^ phenotype and the absence of the deleted region was assayed by PCR with primers that hybridized into the flanking regions of the deletion target (30 cycles of 94 °C during 60 s, 50 °C 30 s, and 72 °C 30 s).

### Promoter activity measurements: determination of luciferase activity

Promoters used to overexpress genes in Se7942 were tested by placing them upstream promoter-less reporter genes *luxAB* from *Photorhabdus luminescens* [74]. The different constructions were introduced into NS1 of Se7942 to test for luciferase activity. Bioluminescence measurements were carried out essentially as described previously [75]. 1.0 ml aliquots were taken at selected time points from shaking cultures and the final OD_720_ was adjusted to 0.5. 100 µl samples were transferred in triplicates to white 96-well microtiter plates (Thermo Fisher Scientific). 100 mM decanal stock solution was prepared in methanol and freshly diluted with BG11 for 2 mM decanal ready-to-use solution prior measuring. 100 µl of 2 mM decanal ready-to-use solution was added to the samples, and the plate was immediately placed into a plate reader (Wallac Victor 2 1420 multilabel counter, PerkinElmer Life Sciences). Bioluminescence was measured for 30 min at 25 °C. The maximum light emission (generally around 10 min after start measuring) was used as the bioluminescence value and normalized to the OD_720_. Results were presented in relative bioluminescence units. Experiments were carried out in triplicates.

### Fatty acid analysis

Total FA content of cyanobacterial cultures was determined by gas chromatography flame ionization detection (GC-FID) analysis. Cultured cells (60 ml) were harvested by centrifugation 24 h post-induction. Preparation of FA methyl esters (FAMEs) was done according to [76]. Briefly, saponification was conducted by adding to each sample 1 ml of saponification reagent (45 g NaOH, 150 ml methanol, 150 ml deionized H_2_0). Samples were vortexed for 10 s, heated 5 min at 100 °C, vortexed 10 s, and heated again, for 25 min. Tubes were then rapidly cooled to room temperature. Methylation was accomplished by adding 2 ml of methylation reagent (325 ml 6 N HCl, 275 ml methanol), vortexing for 10 s, and heating for 10 min at 80 °C. After cooling to room temperature, FAMEs were removed from the acidic aqueous phase and transferred to an organic phase by adding 1.25 ml extraction solvent (hexane:methyl tert-butyl ether [MTBE] 1:1), mixing end-over-end in a laboratory rotator for 10 min, and removing the lower aqueous phase. The organic phase was washed in 3 ml base wash (10.8 g NaOH, 900 ml distilled water) for 5 min with end-over-end mixing. Two-thirds of the upper solvent phase were removed for FAME analysis. Samples were stored at − 20 °C. FAME analysis by GC–FID employed an Agilent 7890A gas chromatograph equipped with a capillary column DB-23 (Agilent Technologies, Santa Clara, CA, USA). The initial oven temperature was set at 130 °C and programmed up to 215 °C at a rate of 2.75 °C/min, and up to 230 °C at a rate of 40 °C/min. The injector temperature was set at 270 °C, and the FID detector at 280 °C. FAMEs were identified by comparing the retention time with Supelco^®^ 37 component FAME standard mix (Sigma-Aldrich, St. Louis, MO, USA).

### Statistical analysis

All measurements were carried out at least in triplicates. Data were expressed as the mean ± SD or mean + SD of n separate experiments, depending on data representation. The significance of differences between groups was evaluated by one-way ANOVA followed by Dunnett’s multiple comparison test or by an unpaired Student’s *t* test. Statistical analyses were carried out using GraphPad Prism software (GraphPad Software Inc., San Diego, CA). Differences were considered statistically significant at *p* < 0.05.

## Additional files

**Additional file 1: Figure S1.** PCR analysis of the *fabH* mutants. Lane 1, Hyperladder I (BioLine); lanes 2–6, MSM23 colonies; lanes 7–12, MSM34 colonies; lanes 13–18, MSM35 colonies. Primers 111 and 112 were used to check for the presence of *fabH* in the mutant strains. The largest band ( ~2.6 kb) belongs to the chromosome copies with *fabH* deletion and the smallest band (~ 2.1 kb) to the wt chromosome copies. **Figure S2.** Luciferase activity in the reporter Se7942 strains. (A) Activity of the P*nrsB* promoter in strain MSM18 with/without adding 5 µM NiSO_4_. (B) Activity of the P*trc* promoter in strain MSM25 induced with different IPTG concentrations. (C) Activity of the P*nrsB* promoter with the theophylline riboswitch in strain MSM22 with/without adding 5 µM NiSO4 and with/without adding 2 mM theophylline. In all cases, data represent the mean ± SD of three individual experiments. **Figure S3.** Construction of the *fadD*-deficient mutant, MSM42, using the system designed by Matsuoka et al. [28]. (A). Scheme of the *fadD* vicinity in wt and mutant strains. Relevant genes are indicated by colored arrows, while USDT and DSDT by boxes. Primers 109 and 110 were used for PCR analysis and are indicated by black arrows. (B). PCR analysis of the *fadD* region. Lane 1, Hyperladder I (BioLine); lanes 2–16, MSM42 colonies; lane 17, wt Se7942 strain (WT). The smallest band (~ 2.3 kb) belongs to the chromosome copies with the *fadD* deletion while the largest band (~ 2.8 kb) corresponds to the wt chromosome copies.

**Additional file 2.** Overview of plasmid constructions.

**Additional file 3: Table S1.** Plasmids used in this study. **Table S2.** Oligonucleotides used to clone genes in this study. **Table S3.** Oligonucleotides used to verify mutants in this study.

## Abbreviations

FA: fatty acid
Se7942: *Synechococcus elongatus* PCC 7942
Ss7002: *Synechococcus* sp. PCC 7002
ALA: alpha-linolenic acid
FAS: fatty acid synthase
*fab*: fatty acid biosynthesis
ACP: acyl-carrier protein
MCAT: malonyl-CoA ACP transacylase
NS1: neutral site 1
KAS: β-ketoacyl-ACP synthase
wt: wild type
Ss6803: *Synechocystis* sp. PCC 6803
MCFA: medium-chain fatty acid
LCFA: long-chain fatty acid
RB-TnSeq: random barcode transposon site sequencing
USDT: upstream sequences of the deletion target
DSDT: downstream sequences of the deletion target
GC–FID: gas chromatography–flame ionization detection
FAME: fatty acid methyl ester
ANOVA: analysis of variance
NS2: neutral site 2
