# Supplementary figures and images for: Engineering the fatty acid synthesis pathway in Synechococcus elongatus PCC 7942 improves omega-3 fatty acid production
Source: Biotechnol Biofuels. 2018 Sep 5;11:239. doi: 10.1186/s13068-018-1243-4 (PMC6123915; doi:10.1186/s13068-018-1243-4)

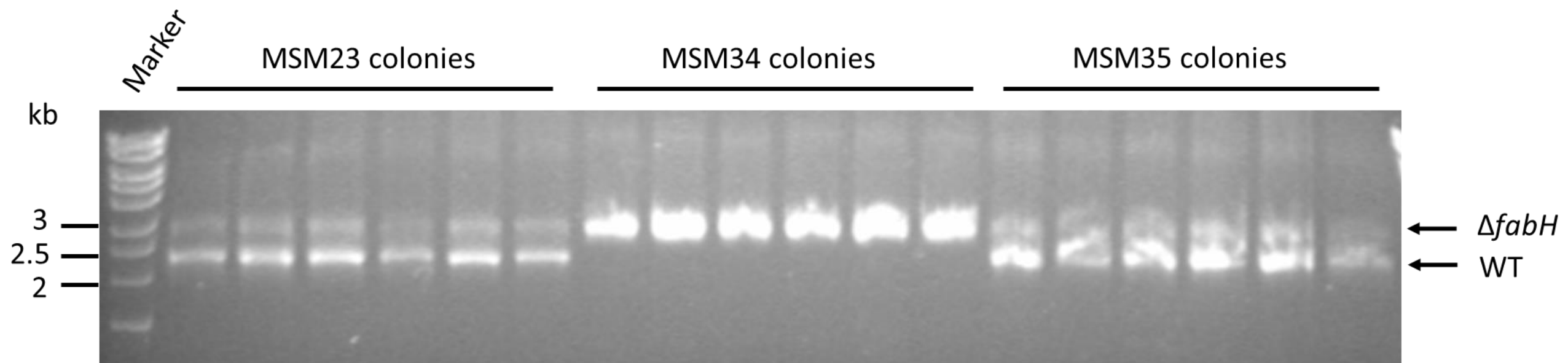

**A**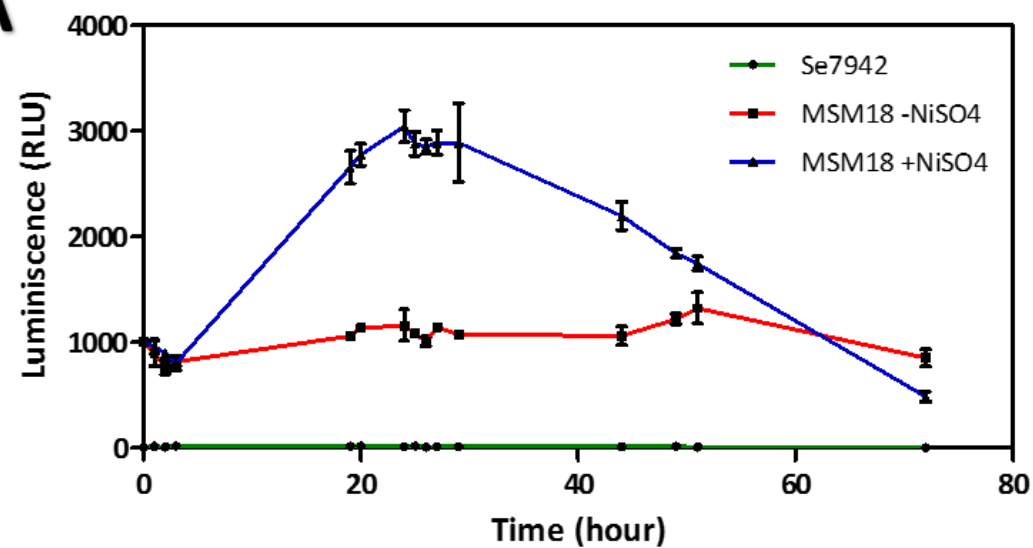**B**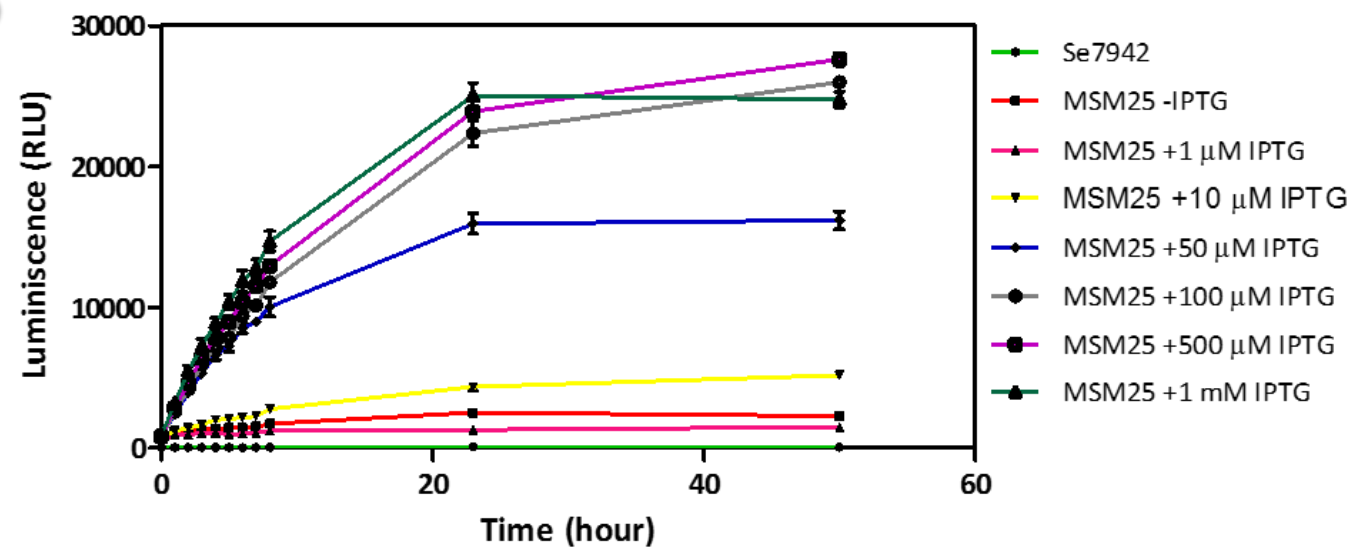**C**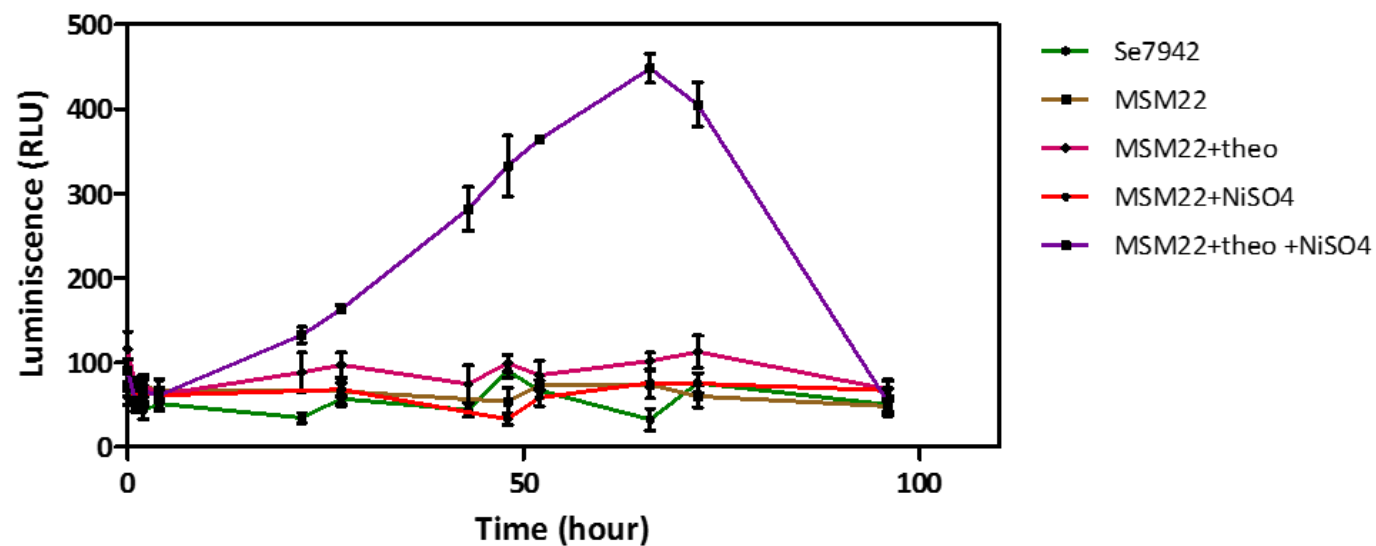

**A**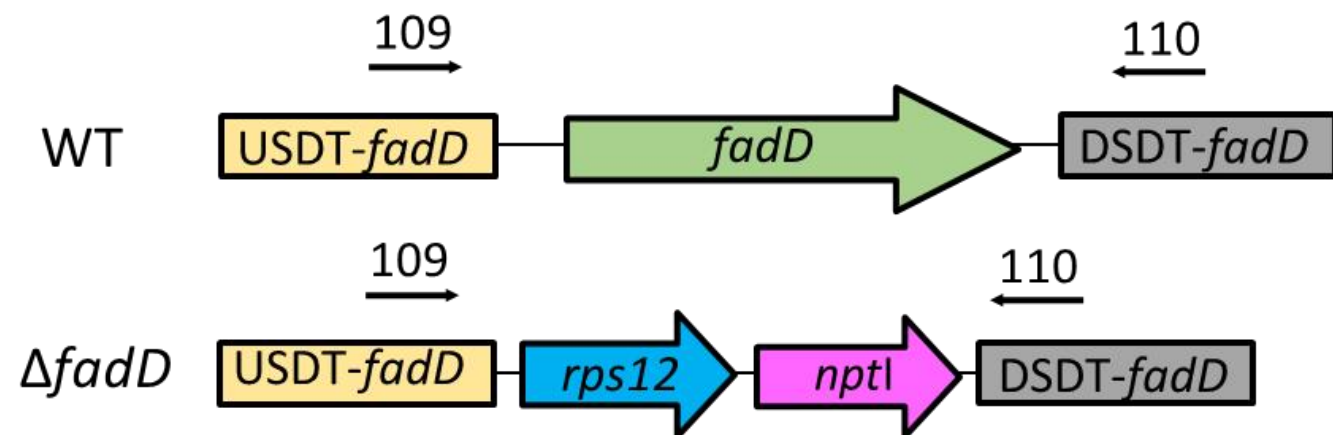**B**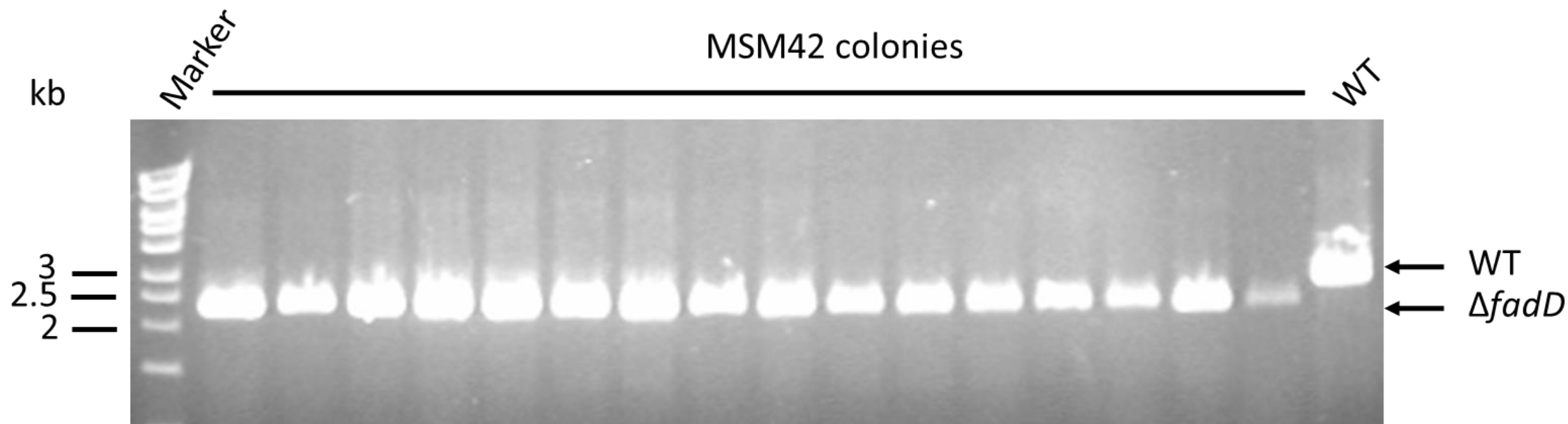

Supplement: Supplementary file 1 — Additional file 1: Figure S1. PCR analysis of the fabH mutants. Lane 1, Hyperladder I (BioLine); lanes 2–6, MSM23 colonies; lanes 7–12, MSM34 colonies; lanes 13–18, MSM35 colonies. Primers 111 and 112 were used to check for the presence of fabH in the mutant strains. The largest band ( ~2.6 kb) belongs to the chromosome copies with fabH deletion and the smallest band (~ 2.1 kb) to the wt chromosome copies. Figure S2. Luciferase activity in the reporter Se7942 strains. (A) Activity of the PnrsB promoter in strain MSM18 with/without adding 5 µM NiSO4. (B) Activity of the Ptrc promoter in strain MSM25 induced with different IPTG concentrations. (C) Activity of the PnrsB promoter with the theophylline riboswitch in strain MSM22 with/without adding 5 µM NiSO4 and with/without adding 2 mM theophylline. In all cases, data represent the mean ± SD of three individual experiments. Figure S3. Construction of the fadD-deficient mutant, MSM42, using the system designed by Matsuoka et al. [28]. (A). Scheme of the fadD vicinity in wt and mutant strains. Relevant genes are indicated by colored arrows, while USDT and DSDT by boxes. Primers 109 and 110 were used for PCR analysis and are indicated by black arrows. (B). PCR analysis of the fadD region. Lane 1, Hyperladder I (BioLine); lanes 2–16, MSM42 colonies; lane 17, wt Se7942 strain (WT). The smallest band (~ 2.3 kb) belongs to the chromosome copies with the fadD deletion while the largest band (~ 2.8 kb) corresponds to the wt chromosome copies. [file 13068_2018_1243_MOESM1_ESM.pdf]
