# Supplementary material for: Engineering the fatty acid synthesis pathway in Synechococcus elongatus PCC 7942 improves omega-3 fatty acid production
Source: Biotechnol Biofuels. 2018 Sep 5;11:239. doi: 10.1186/s13068-018-1243-4 (PMC6123915; doi:10.1186/s13068-018-1243-4)
Supplement: Supplementary file 2 — Additional file 2. Overview of plasmid constructions. [file 13068_2018_1243_MOESM2_ESM.docx]

**Plasmid constructions**

All plasmids used in this work are listed in Table S1 (see Additional file 3). Primers used to clone genes and verify the mutant strains are respectively listed in Table S2 and S3 (see Additional file 3). Unless otherwise indicated, the constructions were built by the Gibson assembly method [[1](#_ENREF_1)]. All constructions were checked by PCR and DNA sequencing.

Genes encoding the alpha and beta luciferase subunits, *luxA* and *luxB*, were amplified from plasmid DNA of pSU2007::Tn*lux*. The Δ12 and Δ15 desaturase genes *desA* and *desB*, as well as the *fabB* gene, encoding the β-ketoacyl-ACP synthase I, were amplified from genomic DNA of Ss7002 colonies. The malonyl-CoA ACP transacylase, β-ketoacyl-ACP synthase II and III genes *fabD*, *fabF*, and *fabH* were amplified from genomic DNA of Se7942 colonies.

To analyze the activity of the P*nrsB* and P*trc* promoters, they were linked to the *luxAB* reporter genes of *Photorhabdus luminescens*, rendering plasmids pMSM51 and pMSM197, respectively. To build pMSM51, the *luxAB* genes and pSyn_1/D-TOPO® were amplified by PCR using primer pairs 21-22 and 23-24, respectively. To generate pMSM197, the *luxAB* genes were amplified using primers 57 and 58 and assembled to the pUAGC280 amplicon obtained with primers 59 and 60. To analyze the activity of the P*nrsB* promoter with the riboswitch in the presence of theophylline, plasmid pMSM134 was generated by intramolecular ligation of a PCR amplicon obtained using phosphorylated primers 45 and 46. To build pMSM236, the *luxAB* genes and pMSM134 fragments amplified by PCR respectively using primer pairs 47-48 and 49-50, were assembled.

To overexpress desaturase genes *desA* and *desB* of Ss7002 (GenBank Accession Number: NC_010475) plasmids pMSM14, pMSM16 and pMSM201 were constructed. Plasmid pMSM14 encodes the *desA* gene (SYNPCC7002_A2756) under the P*nrsB* promoter. For its construction, gene *desA* was amplified using primers 13 and 14, digested with *Eco*RI and *Nde*I, followed by ligation to pSyn_1/D-TOPO®, previously digested with the same enzymes. Plasmid pMSM16 encodes the *desA* (SYNPCC7002_A2756) and *desB* (SYNPCC7002_A0159) genes under the P*nrsB* promoter. To construct pMSM16, gene *desB* was amplified using primers 15 and 16. The resulting PCR product and pMSM14 were digested with *Not*I and *Nde*I and ligated. Plasmid pMSM201 contains *desA* and *desB* desaturase genes under the P*trc* promoter. Primer pairs 61-14 and 15-62 were used to amplify *desA* and *desB* from Ss7002, respectively. The *desA* and *desB* amplicons were respectively cloned in the *Eco*RI/*Not*I and *Not*I/*BamH*I sites of pUAGC280.

Two plasmids were constructed to modify the expression of the *fabH* gene of Se7942 (Synpcc7942_1455; GenBank Accession Number: NC_007604) by overexpression from the *Ptrc* promoter (pMSM228) or deletion (pMSM182). Gene *fabH* was amplified by PCR using primer pair 67-68, digested with *Eco*RI and *Sal*I, and ligated to pUAGC280 to produce pMSM228. Plasmid pMSM182 was generated in two steps. Firstly, the upstream sequence of the deletion target (USDT) of the *fabH* gene was amplified with primers 51 and 52 and linked to the pDEP52 amplicon generated with primers 9 and 10. Secondly, the downstream sequence of the deletion target (DSDT) of the *fabH* gene was amplified using primer pair 53-54, restricted with *Xba*I/*Not*I and cloned in the *Spe*I/*Not*I sites of pMSM176 to generate pMSM182.

The *fabB* gene of Ss7002 (SYNPCC7002_ A1002; GenBank Accession Number: NC_010475) was cloned under two different promoters, P*nrsB* (pMSM90) and *Ptrc* (pMSM202). To build pMSM90 *fabB* was amplified using primers 35 and 36, digested with *Eco*RI and *Nde*I, and cloned in the same sites of pSyn_1/D-TOPO®. To build pMSM202 *fabB* was amplified using primers 63 and 64, digested with *Eco*RI and *Bam*HI, and cloned in the same sites of pUAGC280.

To overexpress the *fabF* gene of Se7942 (Synpcc7942_0537; GenBank Accession Number: NC_007604) in two neutral chromosomal regions, NS1 and NS2, plasmids pMSM234 and pMSM253 were constructed. Gene *fabF* was amplified with primer pair 70-71, digested with *Eco*RI and *BamH*I, and ligated to the same sites of pUAGC280, producing plasmid pMSM234. The *luxAB* genes and the T4 terminator were removed from pAM1580 by intramolecular ligation of a PCR amplicon obtained using phosphorylated primers 74 and 75, producing pMSM249. This plasmid was used in turn to clone the P*trc*::*fabF* fragment, which was amplified from pMSM234 plasmid DNA using primers 76 and 78. The amplicon was digested with *Spe*I/*Kpn*I and cloned in the same sites of pMSM249, rendering plasmid pMSM253.

For overexpressing the *fabD* gene of Se7942 (Synpcc7942_1456; GenBank Accession Number: NC_007604), it was amplified using primers 55 and 56, digested with *Eco*RI and *Bam*HI, and ligated to the same sites of pUAGC280 to produce pMSM196.

To delete the *fadD* gene of Se7942 (Synpcc7942_0918; GenBank Accession Number: NC_007604), its DSDT and USDT regions were amplified by using primer pairs 1-2 and 81-82. The DSDT amplicon was linked to a pDEP52 fragment generated by PCR using primers 3 and 4 to produce pMSM1. Subsequently, the USDT amplicon was linked to a pMSM1 fragment amplified by PCR using primers 9 and 10, producing pMSM266.

**References**

1. Gibson DG, Young L, Chuang RY, Venter JC, Hutchison CA, 3rd, Smith HO. Enzymatic assembly of DNA molecules up to several hundred kilobases. Nat Methods. 2009;6 5:343-345.
