# Supplementary material for: Engineering the fatty acid synthesis pathway in Synechococcus elongatus PCC 7942 improves omega-3 fatty acid production
Source: Biotechnol Biofuels. 2018 Sep 5;11:239. doi: 10.1186/s13068-018-1243-4 (PMC6123915; doi:10.1186/s13068-018-1243-4)
Supplement: Supplementary file 3 — Additional file 3: Table S1. Plasmids used in this study. Table S2. Oligonucleotides used to clone genes in this study. Table S3. Oligonucleotides used to verify mutants in this study. [file 13068_2018_1243_MOESM3_ESM.docx]

Table S1. Plasmids used in this study.

| Plasmid | Relevant genotype^a^ | Source or reference | Primers to check plasmids^b^ |
| --- | --- | --- | --- |
| pSB1C3 | Rep(pMB8); Cm^r^; backbone for BioBrick parts cloning | http://parts.igem.org/Part:pSB1K3 |  |
| pSyn_1/D-TOPO® | NS1 targeting vector; P*nrsB*; Sp^r^ | GeneArt, Invitrogen |  |
| pUAGC280 | NS1 targeting vector with *lacI^q^* and P*trc*; Ap^r^ Sm^r^ | [[1](#_ENREF_1)] |  |
| pAM1580 | NS2 targeting vector carrying *luxAB* reporter genes; Cm^r^ Ap^r^ | [[2](#_ENREF_2)] |  |
| pSU2007::Tn*lux* | pSU2007::*luxCDABE*, IncW, Tp^r^ Km^r^ Gm^r^ | [[3](#_ENREF_3)] |  |
| pDEP52 | pSB1C3::(*rps12*-*npt*I); Km^r^ Cm^r^ | [[4](#_ENREF_4)] |  |
| pMSM1 | pDED52::DSDT-*fadD*; Km^r^ Cm^r^ | This work | 98-110 |
| pMSM14 | pSyn_1/D-TOPO®::*desA*; Sp^r^ | This work | 100-101 |
| pMSM16 | pMSM14:(*desA-desB*); Sp^r^ | This work | 100-101 |
| pMSM51 | pSyn_1/D-TOPO®::*luxAB*; Sp^r^ | This work | 104-105 |
| pMSM90 | pSyn_1/D-TOPO®::*fabB*; Sp^r^ | This work | 100-101 |
| pMSM134 | pSyn_1/D-TOPO® with riboswitch and RBS described by Nakahira et al. [[5](#_ENREF_5)]; Sp^r^ | This work | 100-101 |
| pMSM142 | pMSM134::*luxAB*; Sp^r^ | This work | 100-101 |
| pMSM176 | pDED52::USDT-*fabH*; Km^r^ Cm^r^ | This work | 97-109 |
| pMSM182 | pDED176::DSDT-*fabH*; Km^r^ Cm^r^ | This work | 98-110 |
| pMSM196 | pUAGC280::*fabD*; Sp^r^ | This work | 104-105 |
| pMSM197 | pUAGC280::*luxA*B; Sp^r^ | This work | 104-105 |
| pMSM201 | pUAGC280::(*desA-desB*); Sp^r^ | This work | 100-101 |
| pMSM202 | pUAGC280::*fabB*; Sp^r^ | This work | 104-105 |
| pMSM228 | pUAGC280::*fabH*; Sp^r^ | This work | 104-105 |
| pMSM234 | pUAGC280::*fabF*; Sp^r^ | This work | 104-105 |
| pMSM236 | pMSM134::*fabH*; Sp^r^ | This work | 100-101 |
| pMSM249 | pAM1580::Δ*luxAB*; Cm^r^ Ap^r^ | This work | 106-108 |
| pMSM253 | pMSM249::(P*trc*-*fabF*); Cm^r^ Ap^r^ | This work | 104-105 |
| pMSM266 | pMSM1::USDT-*fadD*; Km^r^ Cm^r^ | This work | 97-109 |

^a^Ap^r^, ampicillin resistance; Sp^r^, spectinomycin resistance; Sm^r^, streptomycin resistance; Cm^r^, chloramphenicol resistance; Km^r^, kanamycin resistance; Tp^r^, trimethoprim resistance; Gm^r^, gentamicin resistance; DSDT, downstream sequences of the deletion target; USDT, upstream sequences of the deletion target.

^b^Primer pairs used to check the constructions by PCR and DNA sequencing. The sequence of these primers is detailed in Table S3 (see Additional file 3).

Table S2. Oligonucleotides used to clone genes in this study.

| Number | Sequence (5’ → 3’)^a^ |
| --- | --- |
| 1 | *TAAATTGCAGTTTCATTTGATGCTCGATGAGTTTTTCTA****A*AATGACCCTCGGTACTCCTCTGCAGC** |
| 2 | *GGAATTGCAGCTAAAACCGGCAGCATT***GCAGGCTTCCTCGCTCACTGACTCGCTGCGCTCGGTCGTT** |
| 3 | **GCAGGCTTCCTCGCTCACTGAC** |
| 4 | **CATTTGATGCTCGATGAGTTTTTCTAA** |
| 9 | **TAGGGGGTTGCTCCTACGCTC** |
| 10 | **GAAATCATCCTTAGCGAAAGCCTATTTTTATAG** |
| 13 | TTCGAATTCGAAGGAGCCCTTCACC**ATGACATCAGTTACTGTGCGC** |
| 14 | TTCCATATG*GCGGCCGC*GTACGTACACCCTT**TTAGCGTTTTTGATGTTGCG** |
| 15 | TACGCGGCCGCGAAGGAGCCCTTCACC**ATGCAAAGTACAGTCCGCTCC** |
| 16 | ATGCATATGGTACGTACACCCTT**TTATTTTTTCGGCTGGTAATACAAG** |
| 21 | *GAGGTGGTGTGAAGCTTCGAATTCGAAGGAGCCCTTCACC***ATGAAATTTGGAAACTTTTTGCTTAC** |
| 22 | *CTGCCGCCAGGCACTCGATGCATATGGTACGTACACCCTT***CTACATGTGGTACTTTTTAATATTATCATC** |
| 23 | **AAGGGTGTACGTACCATATGCATCG** |
| 24 | **GGTGAAGGGCTCCTTCGAATTC** |
| 35 | TTCGAATTCGAAGGAGCCCTTCACC**ATGGTGCGGGTAGTGATAACGG** |
| 36 | TTCCATATGGTACGTACACCCTT**TCAAAAATATTTTTGAAGGGCGATC** |
| 45 | TGGCAGCACCCTGCTAAGGAGGCAACAAG**GGATCCACCATATGCATCGAGTGCCTGG** |
| 46 | AGGGCATCAAGACGATGCTGGTATCACCC**GAATTCGAAGCTTCACACCACCTC** |
| 47 | *GCCCTTGGCAGCACCCTGCTAAGGAGGCAACAAGGGATCC***ATGAAATTTGGAAACTTTTTGCTTACATAC** |
| 48 | *ACCACCGCGCTACTGCCGCCAGGCACTCGATGCATATGGT***TTAGGTATATTCCATGTGGTACTTCTTAATATTATC** |
| 49 | ACCATATGCATCGAGTGCCTGG |
| 50 | GATCCCTTGTTGCCTCCTTAGCAG |
| 51 | *CATTAACCTATAAAAATAGGCTTTCGCTAAGGATGATTTC***ATGACGCGTGCGCGGATC** |
| 52 | *GAAGACGCTAAATCCAGCTGAGCGTAGGAGCAACCCCCTA***CTAGCTCACCACTGGCTCAGTCACC** |
| 53 | GTTTCTTCGAATTCGCGGCCGCTTCTAGAG**ATGGCTAAAACGGTGTGGGTGTTTC** |
| 54 | GTTTCTTCCTGCAGCGGCCGCTACTAGTA**TTAGACCGTGAGGTCTGCTGCTGTTC** |
| 55 | CATGGAATTC**ATGGCTAAAACGGTGTGGGTG** |
| 56 | TAGAGGATCC**TTAGACCGTGAGGTCTGCTGCTG** |
| 57 | *AACAATTTCACACAGGAAACAGACCATGGAATTC***ATGAAATTTGGAAACTTTTTGCTTAC** |
| 58 | *CCCCCCCTCGAGGTCGACTCTAGAGGATCC***CTACATGTGGTACTTTTTAATATTATCATC** |
| 59 | **GGATCCTCTAGAGTCGACCTCG** |
| 60 | **GAATTCCATGGTCTGTTTCCTG** |
| 61 | CATGGAATTC**ATGACATCAGTTACTGTGCGC** |
| 62 | TAGAGGATCC**TTATTTTTTCGGCTGGTAATACAAG** |
| 63 | CATGGAATTC**ATGGTGCGGGTAGTGATAACGG** |
| 64 | TAGAGGATCC**TCAAAAATATTTTTGAAGGGCGATC** |
| 67 | CATGGAATTC**TTGACTCGACCTGGCGTTGG** |
| 68 | CGAGGTCGAC**CTAAACCACCGTGCCCCAAC** |
| 70 | CATGGAATTC**ATGACTGAAACCGGACGCCAG** |
| 71 | TAGAGGATCC**CTAGGGATGGAATTTCCGGAAGG** |
| 72 | TTCGGTACC**TTGACTCGACCTGGCGTTGG** |
| 73 | TTCCATATG**CTAAACCACCGTGCCCCAAC** |
| 74 | TGGGGTACCGGCCGCGGCCGC**AACATTTCCTTATTTGTTGGTATTACGATAAG** |
| 75 | CCTACTAGTGGCCATTTAAAT**ACGTTTAACTGATGCTGAAGGGG** |
| 76 | GGCCACTAGT**TTGACAATTAATCATCCGGCTCG** |
| 78 | GGCCGGTACC**CTAGGGATGGAATTTCCGGAAGG** |
| 81 | *CATTAACCTATAAAAATAGGCTTTCGCTAAGGATGATTTC***AGCTTGGGTCGATGCCCG** |
| 82 | *GAAGACGCTAAATCCAGCTGAGCGTAGGAGCAACCCCCTA***TCGGTCTACTCTCCTCAGCCAGC** |

^a^Overlapping sequences to perform the Gibson assembly are in italics. Sequences hybridizing to the template DNA are in bold. Restriction sites are underlined.

Table S3. Oligonucleotides used to verify mutants in this study.

| Number | Sequence (5’ → 3’) |
| --- | --- |
| 97 | TGCCACTTGACGTCTAAGAA |
| 98 | ATTACCGCCTTTGAGTGAGC |
| 100 | AGTCGGCAAATAACCCTCGG |
| 101 | GCCAGGCATCAAATAAAACG |
| 102 | GCTTGCCTTCCTATGGTTCGG |
| 103 | CCTGTGCAGCAGGAGCGG |
| 104 | GTGGACCGCTTGCTGCAACTC |
| 105 | GCTTGGCAGACCGCTGGTG |
| 106 | CGCTTCCCACGCTGAGAGG |
| 108 | TGCTGGGTAGTTCTCCGCTGC |
| 109 | CAAAAAGCCCAACTCCGCCC |
| 110 | CCGATACCAGGATCTTGCCATCC |
| 111 | GTCGGCTTGGTCAACATCGGC |
| 112 | CACTACGCCTTCGGTGTTGGC |

**References**

1. Moronta-Barrios F, Espinosa J, Contreras A. Negative control of cell size in the cyanobacterium *Synechococcus elongatus* PCC 7942 by the essential response regulator RpaB. FEBS Lett. 2013;587 5:504-509.

2. Andersson CR, Tsinoremas NF, Shelton J, Lebedeva NV, Yarrow J, Min H, Golden SS. Application of bioluminescence to the study of circadian rhythms in cyanobacteria. Methods Enzymol. 2000;305:527-542.

3. Fernandez-Lopez R, Machon C, Longshaw CM, Martin S, Molin S, Zechner EL, Espinosa M, Lanka E, de la Cruz F. Unsaturated fatty acids are inhibitors of bacterial conjugation. Microbiology. 2005;151 Pt 11:3517-3526.

4. Encinas D: DNA transactions in a model cyanobacterium *Synechococcus elongatus* PCC 7942. University of Cantabria, Instituto de Biomedicina y Biotecnología de Cantabria (Universidad de Cantabria—Consejo Superior de Investigaciones Científicas); 2014.

5. Nakahira Y, Ogawa A, Asano H, Oyama T, Tozawa Y. Theophylline-dependent riboswitch as a novel genetic tool for strict regulation of protein expression in Cyanobacterium *Synechococcus elongatus* PCC 7942. Plant Cell Physiol. 2013;54 10:1724-1735.
